# Prevalence and associated characteristics of anti-SARS-CoV-2 antibodies in Mexico 5 months after pandemic arrival

**DOI:** 10.1186/s12879-021-06550-5

**Published:** 2021-08-19

**Authors:** Cristina Díaz-Salazar, Adriana Sánchez-García, René Rodríguez-Gutiérrez, Adrián Camacho-Ortiz, Donato Saldívar-Rodríguez, José Gerardo González-González

**Affiliations:** 1 Municipal Health Clinic of Guadalupe, Av. Benito Juárez 928, Colonia Nueva Exposición, 67150 Guadalupe, Nuevo Leon Mexico; 2 Facultad de Medicina y Hospital Universitario “Dr. José E. González”, Endocrinology Division, Department of Internal Medicine, Universidad Autónoma de Nuevo León, Av. Madero y Av. Gonzalitos S/N, Colonia Mitras Centro, 64460 Monterrey, Nuevo Leon Mexico; 3 Plataforma INVEST Medicina UANL-KER Unit Mayo Clinic (KER Unit Mexico), Calle Dr. Eduardo Aguirre Pequeño S/N Edificio CRIDS, Colonia Mitras Centro, 64460 Monterrey, Nuevo Leon Mexico; 4 Knowledge and Evaluation Research Unit in Endocrinology, Mayo Clinic, 210 2nd St SW, Rochester, MN 55905 USA; 5 Facultad de Medicina y Hospital Universitario “Dr. José E. González”, Infectious Diseases Department, Universidad Autónoma de Nuevo León, Av. Madero y Av. Gonzalitos S/N, Colonia Mitras Centro, 64460 Monterrey, Nuevo Leon Mexico; 6 Facultad de Medicina y Hospital Universitario “Dr. Jose E. González”, Obstetrics Division, Universidad Autónoma de Nuevo León, Av. Madero y Av. Gonzalitos s/n, Colonia Mitras Centro, 64460 Monterrey, Nuevo Leon Mexico

**Keywords:** COVID-19, Prevalence, Survey, Serology, Anti-SARS-CoV-2, Mexico

## Abstract

**Background:**

Seroprevalence of anti-SARS-CoV-2 antibodies is now available in several world regions to better estimate transmission dynamics. However, to date, there is no epidemiological data regarding anti-SARS-CoV-2 prevalence in Mexico. Therefore, we aimed to determine the prevalence of anti-SARS-CoV-2 antibodies and define the clinical and demographic characteristics associated with seroprevalence.

**Methods:**

We conducted a cross-sectional serological survey in Ciudad Guadalupe, NL, Mexico. City government employees voluntarily participated during July 2020. Demographic and clinical characteristics were collected at the time of blood sampling to analyze the associated characteristics. IgM/IgG antibodies were determined using a qualitative chemiluminescent immunoassay. Descriptive statistics were used for categorical and continuous variables. Statistical significance was tested using the Chi-squared test, Student’s t-test and the Mann–Whitney. Logistic regression models and the odds ratios (adjusted and unadjusted) were used to estimate the association of demographic and clinical characteristics.

**Results:**

Of the 3,268 participants included, 193 (5.9%, 95% CI 5.1–6.8) tested positive for IgM/IgG against SARS-CoV-2. Sex, city of residence, and comorbidities did not show any association with having IgM/IgG antibodies. A total of 114 out of 193 (59.1%) subjects with a positive test were asymptomatic, and the odds of being positive were higher in those who reported symptoms of COVID-19 in the previous four weeks to the survey (OR 4.1, 95% CI 2.9–5.5).

**Conclusions:**

There is a low rate of SARS-CoV-2 infection among government employees that have continuously been working during the pandemic. Six in ten infections were asymptomatic, and seroprevalence is low and still far from herd immunity. Epidemiological surveillance and preventive measures should be mandatory.

## Background

Since the first case reported of the SARS-CoV-2 virus was reported in Mexico (February 27, 2020) to date (August 30th, 2020), there are 595,841 confirmed cases and 64,158 deaths [1]. Nuevo Leon, a northern state comprising a population of 5,119,504 inhabitants and which includes one out of the three most populated metropolitan areas in Mexico, has registered 49,457 confirmed cases and 2,403 deaths (August 30th, 2020), placing it in the third position nationwide [2]. Despite the increasing epidemiological surveillance efforts using the gold-standard quantitative polymerase chain reaction (PCR), testing is limited to symptomatic individuals, and it varies widely according to the test availability, even in the same country. Moreover, the occurrence of mild or asymptomatic infections leads to an underestimation of cases. Thus, seroepidemiological studies will allow better estimates of transmission dynamics, cumulative prevalence, and the proportion of the remaining susceptible populations [3].

Seroprevalence of anti-SARS-CoV-2 antibodies is now available in several world regions. In Europe, firstly affected by SARS-CoV-2, Germany, Spain, and Switzerland determined a 1.22% (March–May 2020), 4.6% (May 2020), and 4.8% (April–May 2020) antibody prevalence, respectively [4–6]. In the Americas, a 2.8% seroprevalence was reported in the state of California, United States, during April 2020, while 0.22% was observed in Brazil during May 2020 in a population-based survey [7, 8]. However, these serological surveys have been conducted at different epidemic phases in each country, using several immunological assays, sample sizes, and study designs in selected populations (population-based, blood donors, health-care workers) [5, 9, 10]. Thus, information derived from other regions is not a true reflection of the local epidemic's occurrence and transmission. To date, there is no epidemiological data regarding anti-SARS-CoV-2 prevalence in Mexico.

We conducted a cross-sectional serological survey in a population of government employees for the qualitative detection of IgM and IgG antibodies against SARS-CoV-2. We aimed to determine, as a primary endpoint, the prevalence of anti-SARS-CoV-2 antibodies and as a secondary endpoint, we defined the clinical and demographic characteristics associated with seroprevalence in the studied population. These results are now implemented for pandemic management in our regional context.

## Methods

### Study design and participants

We conducted a community level serological survey in Ciudad Guadalupe, the second most populated city (682,880 inhabitants) included in the metropolitan area of Monterrey, Nuevo Leon, Mexico [11]. The participants were drawn from a complete database of 4,220 government employees who were voluntarily invited to participate in this serological survey. Participants were included from July 9 to July 23, 2020. The work was carried out in accordance with Declaration of Helsinki for studies involving humans. Written informed consent was obtained from all participants, according to the research protocol approved by the Institutional Review Board of the School of Medicine and “Dr. Jose E. Gonzalez” University Hospital. Fieldwork was carried out by trained health care professionals in the community health clinic. We obtained demographic (age, sex, zip code, hometown, occupation) and clinical information using a standardized electronic questionnaire focused on symptoms related to coronavirus disease 19 (COVID-19), contact with confirmed cases, and comorbidities. After a 4 h fasting period, blood samples were collected, labeled, and sent to the Metabolic Research Laboratory at the Endocrinology Division of the “Dr. Jose Eleuterio Gonzalez” University Hospital.

### Detection of anti-SARS-CoV-2 antibodies

We selected a qualitative chemiluminescent immunoassay to detect IgM/IgG against SARS-CoV-2 (Elecsys Anti-SARS-CoV-2, Roche, Germany, Ref. 09203079190) using the Cobas e801 automated analyzer (Roche, Germany). The National Health Department approved this test for the diagnostic intended use. The manufacturer reported a 99.5% sensitivity and specificity of 99.8% after 14 days post-PCR confirmation. This test uses a recombinant protein representing the nucleocapsid (N) antigen for the determination of antibodies against SARS‐CoV‐2 in the serum sample. The software automatically determines chemiluminescent emission by comparing the electrochemiluminescence signal obtained from the sample's reaction product with the signal of the cut-off value previously obtained by calibration (COI, cut-off index) using the positive and negative quality controls.

### Statistical analysis

Statistical analysis was performed in the IBM SPSS Statistic software V22 (IBM Corp., USA) and EpiR for the Statistical Analysis of Epidemiology package [12]. The sample size calculated considering a SARS-CoV-2 seroprevalence of 5%, with a precision of ± 2.0% at the 95% confidence intervals, was 608 subjects. We reported the raw frequencies of positive tests as a proportion of the sample size. We compared our estimated cumulative prevalence against the official government PCR-based data of confirmed cases at the time of the survey. Descriptive statistics were used for categorical (frequencies and percentages) and continuous (means and standard deviations) variables. Statistical significance (*P*-value < 0.05) was tested using the Chi-squared and Fisher test for categorical variables, while Student’s *t*-test and the Mann–Whitney test were performed for continuous variables. Logistic regression models and the odds ratios (adjusted and unadjusted) were used to estimate the association of demographic and clinical characteristics identified as statistically significant in the bivariate analysis.

## Results

### Overall study population

A total of 4220 subjects were invited to participate in the serological survey, including 694 retired employees. During the 11 days of fieldwork, we collected 3268 (77.4%) blood samples and clinical data and 952 (22.6%) subjects did not attend the invitation due to planned vacations, sick leave, and work schedule unavailability. Table 1 shows the demographic and clinical data. The median age was 40 years (IQR 31–49), and 61.4% were males. Median BMI was 28.4 kg/m^2^, and obesity and overweight were observed in 38 and 39.4% of the population, respectively. Previously known hypertension and type 2 diabetes were referred by 8.8% of participants for both diseases. Besides, 60.3% declared to live in Ciudad Guadalupe, and police, firefighters, public security, and administrative tasks were the main occupations registered.

**Table 1:** Demographic and clinical characteristics of the population

	Total sample (n = 3268)	Negative IgM/IgG (n = 3075)	Positive IgM/IgG (n = 193)	*P*
Males, n (%)	2008 (61.4)	1891 (61.5)	117 (60.6)	0.82
Age, yrs				0.29
18–34, n (%)	1127 (34.5)	1059 (34.5)	68 (35.2)	
35–49, n (%)	1328 (40.6)	1242 (40.4)	86 (44.6)	
50–65, n (%)	765 (23.4)	730 (23.7)	35 (18.1)	
> 65, n (%)	48 (1.5)	44 (1.4)	4 (2.1)	
Weight^a^, kg	80.0 (69–90)	80.0 (69–90)	82 (72–92)	0.13
Height^a^, m	1.67 (1.6–1.7)	1.67 (1.6–1.7)	1.67 (1.6–1.7)	0.86
BMI^a^, kg/m^2^	28.41 (25.3–32)	28.4 (25.2–32)	29.0 (25.6–32.6)	0.18
BMI categories				0.08
Underweight, n (%)	17 (0.5)	17 (0.6)	0	
Normal, n (%)	721 (22.1)	685 (22.3)	36 (18.7)	
Overweight, n (%)	1289 (39.4)	1209 (39.3)	80 (41.5)	
Obesity I-III, n (%)	1241 (38)	1164 (37.8)	77 (39.8)	
Comorbidities				
Hypertension, n (%)	287 (8.8)	269 (8.7)	18 (9.3)	0.79
Diabetes type 2, n (%)	287 (8.8)	273 (8.9)	14 (7.3)	0.51
Obesity, n (%)	1241 (38)	1164 (37.8)	77 (39.8)	0.59
City				0.08
Guadalupe, n (%)	1970 (60.3)	1846 (60.0)	124 (64.2)	
Monterrey, n (%)	495 (15.1)	257 (8.4)	17 (8.8)	
Juárez, n (%)	274 (8.4)	465 (15.1)	30 (15.5)	
Other, n (%)	530 (16.2)	507 (16.5)	22 (11.5)	
Occupation				0.09
Police, firefighters and public safety, n (%)	1051 (32.2)	982 (31.9)	69 (35.7)	
Office/Management, n (%)	696 (21.2)	660 (21.5)	36 (18.7)	
Maintainance/Janitors, n (%)	608 (18.7)	583 (18.9)	25 (12.9)	
Healt-care workers, n (%)	163 (5)	155 (5)	8 (4.1)	
Other, n (%)	750 (22.9)	695 (22.7)	55 (28.6)	

^a^Median (IQR). *IgG* Type G immunoglobulin, *IgM* Type M immunoglobulin

### Prevalence of anti-SARS-CoV-2 and characteristics of seropositive subjects

We were able to identify anti-SARS-CoV-2 antibodies in 193 out of 3,268 subjects, resulting in a raw prevalence of 5.9% (95% CI 5.1–6.7) during July 2020. Cases with positive IgM/IgG were not different in age, sex, weight, height, or BMI from seronegative subjects. There was a statistical difference in the occupation category of cleaning/maintenance (*P* = 0.04). At the time of the survey, 83.9% of the participants had not experienced COVID-19 symptoms, while 33.7% of the subjects reported a known contact with a confirmed COVID-19 case. As expected, self-reported symptoms during the previous four weeks to the survey were statistically different between the groups (Table 2), including the contact with a confirmed COVID-19 case (*P* = 0.013).

**Table 2:** COVID-19 symptoms during the previous four weeks at the time of the serosurvey

	Seronegative IgM/IgG (n = 3075)	Seropositive IgM/IgG (n = 193)	*P*
Asymptomatic, n (%)	2627 (85.4)	114 (59.1)	< 0.001
Symptomatic, n (%)	448 (14.6)	79 (40.9)	< 0.001
Self-reported symptoms			
Cough, n (%)	119 (3.9)	47 (24.4)	< 0.001
Sore throat, n (%)	202 (6.6)	33 (17.1)	< 0.001
Nasal discharge, n (%)	85 (2.8)	15 (7.8)	0.001
Headache, n (%)	173 (5.6)	32 (16.6)	< 0.001
Anosmia, n (%)	63 (2.0)	40 (20.7)	< 0.001
Muscle/body aches, n (%)	98 (3.2)	31 (16.1)	< 0.001
Diarrhea, n (%)	92 (3.0)	23 (11.9)	< 0.001
Fever, n (%)	62 (2.0)	29 (15.0)	< 0.001
Shortness of breath, n (%)	40 (1.3)	21 (10.9)	< 0.001
Contact with confirmed case, n (%)	416 (13.5)	39 (20.2)	0.013

*COVID-19* Coronavirus disease 2019, *IgG* Type G immunoglobulin, *IgM* Type M immunoglobulin

### Characteristics associated with anti-SARS-CoV-2 seroprevalence

**Fig. 1:**
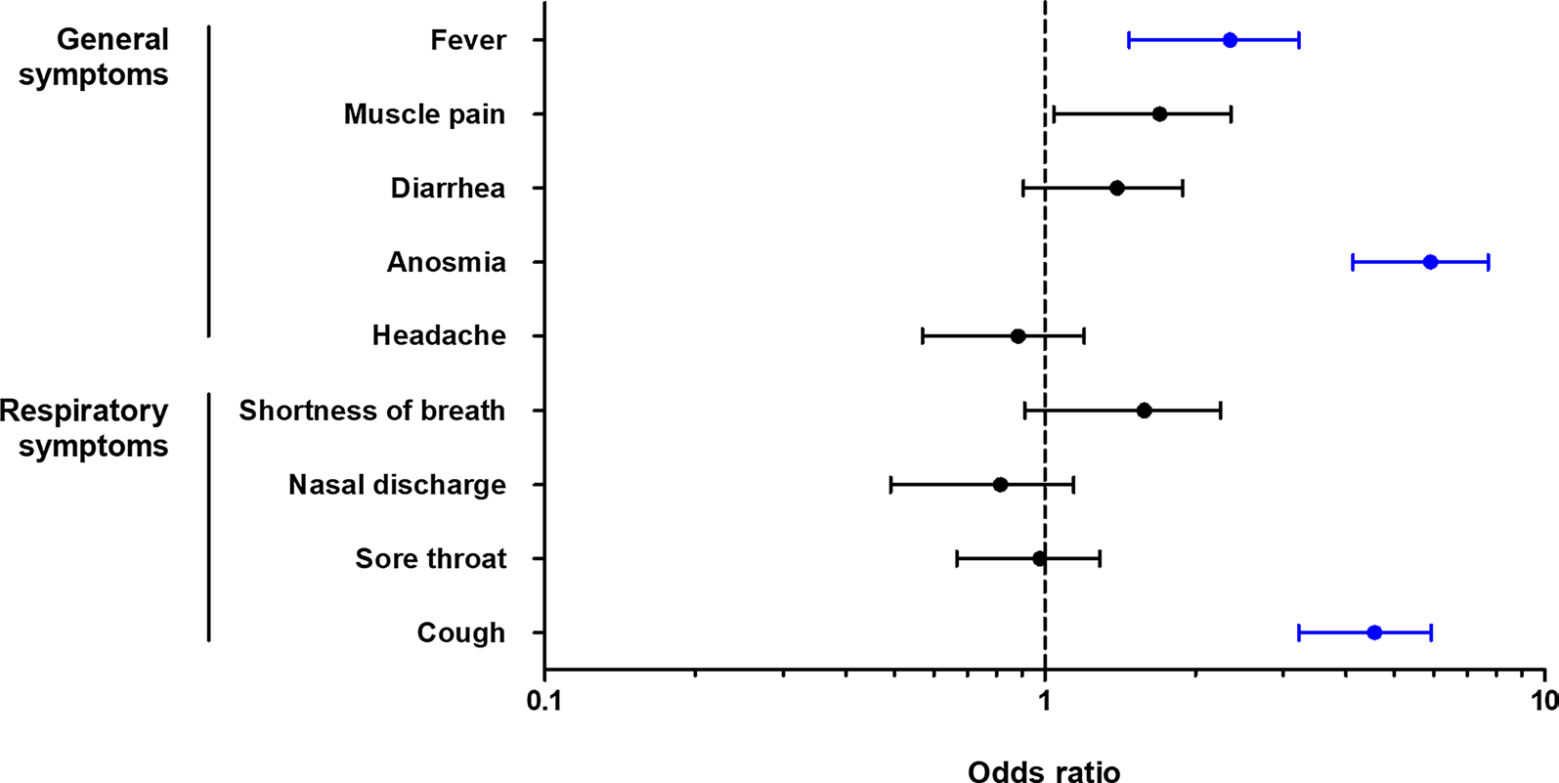
Odds ratios for symptoms associated with positive IgM/IgG against SARS-CoV-2 in the multivariate model. In blue, statistically significant symptoms: anosmia (5.4, 95% CI 3.1–9.2, *P* < 0.001), cough (4.2, 95% CI 2.4–7.1, *P* < 0.001), and fever (2.0, 95% CI 1.02–3.9, *P* = 0.04)

Sex, city of residence, and comorbidities did not show any association with having IgM/IgG antibodies against SARS-CoV-2 (Table 3); while 59.1% (114 out of 193) individuals with a positive test were asymptomatic. The odds of being positive were higher in those who reported COVID-19 symptoms in four weeks before the survey (OR 4.06, 95% CI 2.99–5.51). Anosmia was the most strongly associated symptom with seropositivity (OR 12.62, 95% CI 8.22–19.37), while sore throat was the least (OR 2.93, 95% CI 1.96–4.38). Inversely, being asymptomatic resulted in a 75% less probability of having a positive IgM/IgG test. Multivariable logistic regression analysis revealed that the odds of being positive were higher in those patients with previous symptoms such as cough, anosmia, and fever (Fig. 1). The initial protective effect for the occupation of cleaning/maintenance was no longer observed when adjusted in the multivariate model (OR 0.71, 95% CI 0.46–1.10, *P* = 0.13). Police, firefighters, and public security did not show any association in the univariate or multivariate models.

**Table 3:** Univariate and multivariate analysis of factors associated with positive anti-SARS-CoV-2 antibodies

	IgM/IgG		Univariable			Multivariable		
	Negative	Positive	OR	95% CI	*P*	OR	95% CI	*P*
Sex								
Males, n (%)	1891 (61.5)	117 (60.6)	0.96	0.72–1.30	0.82			
City of residence								
Guadalupe, n (%)	1846 (60.0)	124 (64.2)	1.20	0.89–1.62	0.26			
Occupation								
Police, firefighters & public safety, n (%)	982 (31.9)	69 (35.8)	1.19	0.88–1.61	0.27			
Office/Management, n (%)	660 (21.5)	36 (18.7)	0.84	0.58–1.21	0.41			
Cleaning/Mantainance, n (%)	583 (18.9)	25 (12.9)	0.64	0.41–0.98	0.04	0.71^b^	0.46–1.10	0.13
Health-care workers, n (%)	155 (5.0)	8 (4.1)	0.82	0.40–1.68	0.73			
Other, n (%)	695 (22.7)	55 (28.6)	1.37	0.99–1.89	0.06			
Comorbidities								
Hypertension, n (%)	269 (8.7)	18 (9.3)	1.07	0.65–1.77	0.79			
Diabetes, n (%)	273 (8.9)	14 (7.3)	0.80	0.46–1.40	0.51			
Obesity, n (%)	1164 (37.9)	77 (39.9)	1.09	0.81–1.47	0.59			
Any comorbiditie^a^, n (%)	1481 (48.2)	100 (51.8)	1.16	0.87–1.55	0.34			
COVID-19 symptoms								
Asymptomatic, n (%)	2627 (85.4)	114 (59.1)	0.25	0.18–0.33	< 0.001			
Symptomatic, n (%)	448 (14.6)	79 (40.9)	4.06	2.99–5.51	< 0.001	3.91^b^	2.86–5.35	< 0.001
Cough, n (%)	119 (3.9)	47 (24.4)	7.98	5.49–11.65	< 0.001	4.17^c^	2.50–6.93	< 0.001
Sore throat, n (%)	202 (6.6)	33 (17.1)	2.93	1.96–4.38	< 0.001			
Nasal discharge, n (%)	85 (2.8)	15 (7.8)	2.97	1.68–5.24	0.001			
Shortness of breath, n (%)	40 (1.3)	21 (10.9)	9.26	5.34–16.06	< 0.001			
Headache, n (%)	173 (5.6)	32 (16.6)	3.33	2.21–5.02	< 0.001			
Anosmia, n (%)	63 (2.0)	40 (20.7)	12.62	8.22–19.37	< 0.001	5.75^c^	3.41–9.71	< 0.001
Diarrhea, n (%)	92 (3.0)	23 (11.9)	4.39	2.71–7.12	< 0.001			
Muscle pain, n (%)	98 (3.2)	31 (16.1)	5.81	3.77–8.97	< 0.001			
Fever, n (%)	62 (2.0)	29 (15.0)	8.59	5.38–13.72	< 0.001	2.44^c^	1.30–4.58	0.006
Contact with confirmed case, n (%)	416 (13.5)	39 (20.2)	1.62	1.12–2.34	0.013			

^a^Other cardiovascular, allergic, endocrine, and neurological diseases. ^b^Adjusted for cleaning/maintenance, symptoms, and contact with a confirmed case. ^c^Adjusted for contact with a confirmed case, cleaning/maintenance, sore throat, and headache. *OR* Odds ratio, *CI* Confidence interval

## Discussion

Our findings from this regional cross-sectional seroprevalence study for SARS-CoV-2 indicate that the prevalence of IgM/IgG antibodies against the novel coronavirus is 5.9% in a city of the metropolitan area of Monterrey. This study is the first data available, since the first case registered in February 2020, on the prevalence of anti-SARS-CoV-2 antibodies in our country. Because our survey was designed to obtain information at a local level, regional differences in cultural habits and other factors might be observed against other areas in Mexico. As the third most populated state in the country, Monterrey's metropolitan area was considered a hotspot for epidemic transmission, and our findings may provide additional data to complete epidemiological surveillance.

Several studies have been published since April 2020 regarding the prevalence of anti-SARS-CoV-2 antibodies. Each one provided a snapshot that mirrors time and space-specific circumstances in the different studied populations and the associated characteristics. Our prevalence was higher than those reported in California, United States (April 2020), and Spain and Switzerland during last May 2020 [5, 6], but lower than the 7.3% determined in the 1104 samples from the general population of Stockholm, Sweden at the end of April, three months after the pandemic arrival [13]. Previous data for other regions or states in Mexico are not currently available. Given the asynchrony in COVID-19 transmission in our country, different seroprevalences might be found. Serosurveys are a useful tool to determine the previous exposure to SARS-CoV-2 and better estimate the true number of infections with and without symptoms. Particularly, Mexico has very limited PCR testing based on the sentinel epidemiological model used for epidemiological surveillance. This survey included a large sample, allowing determining an initial reference of previous exposure in asymptomatic individuals. Furthermore, sampling was made in a selected population that belongs to the exposed essential government workforce. Also, demographic characteristics such as age, sex, and city of residence of the sampled population were different from the overall population and may affect generalizability of the results. These characteristics will make it feasible for prospective and follow-up studies. In conjunction, the prevalence of 5.9%, implying that around 40,289 people were infected in Ciudad Guadalupe (population 682,880 in 2015) during July 2020 [11], 10.3 times the average 3,898 PCR confirmed cases at the time of this survey (July 23, 2020) [2]. Notably, Nuevo Leon has the higher rate of PCR test per million people in our country. Similar assumptions could be made for other regions as soon as local data become available.

In agreement with our findings, sex was not associated with anti-SARS-CoV-2 antibodies either in selected populations, health-care workers, and population-based studies. In contrast, age was associated with higher odds of having a positive test in Brazilian blood donors [14], while the opposite was seen in Swiss children and adults > 65 years in a population-based survey [6]. Consistently with our results, no association was observed in the professional category, previous contact with a COVID-19 case, and comorbidities [15]. Although hypertension and type 2 diabetes were associated with a higher risk for poor outcomes in COVID-19 patients [16], no relationship was found with positive IgM/IgG. Educational level, household size, and symptoms were associated with the presence of antibodies [6, 14, 15]. In our study, as expected, previous COVID-19 symptoms were associated with seropositivity (OR 4.06, 95% CI 2.99–5.51, *P* < 0.001), although a higher OR was reported by Garcia-Bateiro et al. (OR 8.8, 95% CI 4.41–17.73, *P* < 0.0001) [15]. Among the symptoms reported, anosmia showed the strongest association in our analysis. Asymptomatic cases (59.1%) compared with other countries were higher than the 32.7%, and 52.4% reported in the general population and health-care workers in Spain, respectively [5, 15]. As much as approximately 80% of infections are expected to be asymptomatic or mild [17].

Recent studies indicate some limitations of antibody testing, besides the inherent diagnostic performance. Although antibodies are expected to correlate with infection and disease presentation, a recent Chinese study showed that some infected individuals did not produce neutralizing humoral evidence of SARS-CoV-2 infection. In 10 (5.7%) out of 175 COVID-19 recovered patients who experienced mild symptoms, neutralizing antibodies against SARS-CoV-2 were unable to cross-react with the SARS-CoV-2 virus [18]. Thus, other immune responses, such as T cells and cytokines, may be involved in recovery. Furthermore, Long e*t al*. reported than 40% of asymptomatic patients became seronegative for IgG in the early convalescent phase [19] suggesting a weaker humoral response that might have implications in the results of serological surveys confounding the true exposure rate.

Our serosurvey has some limitations that may be considered. First, we performed an isolated IgM/IgG test, obtaining a cross-sectional seroprevalence. A prospective and longitudinal survey will be highly valuable in following seroconversion and determining household transmission and lockdown effect in the long-term. Second, children were not included in the survey; additional studies will be needed to determine SARS-CoV-2 spread in this group. Finally, due to the study design, these results represent our sampled population's prevalence estimates and do not necessarily reflect the entire state prevalence. Otherwise, as strengths for the study, we used an appropriate and powered sample size to provide a reference prevalence value. Also, it seems we surveyed at the right epidemic moment (WHO Phase 3). Besides, we used a high-sensitivity chemiluminescent immunoassay, as reported by Muench et al. [20], which has shown a better diagnostic performance than other tests.

## Conclusions

In conclusion, this study provides a regional estimation of SARS-CoV-2 dissemination in the densely populated northern region of our country. Six in ten infections were asymptomatic, and despite the significant impact of COVID-19 in Mexico, seroprevalence is still very low and, more importantly, far from herd immunity. Epidemiological surveillance and preventive measures to avoid transmission, such as social distancing and facial masks use, should still be enforced in the population.

## Data Availability

The datasets generated and analyzed during the current study are available in the Academic Digital Repository (Univesidad Autónoma de Nuevo León), [http://eprints.uanl.mx/19978/].

## Abbreviations

Anti-SARS-CoV-2: Severe acute respiratory syndrome coronavirus 2 antibodies
BMI: Body mass index
CI: Confidence interval
COI: Cut-off index
COVID-19: Coronavirus disease 19
IgM: Immunoglubulin type M
IgG: Immunoglobulin type G
PCR: Polymerase chain reaction
OR: Odds ratio
